# The effect of prolonged G-quadruplex stabilization on the functions of human cells

**DOI:** 10.1038/s41598-025-04791-x

**Published:** 2025-06-04

**Authors:** Nargis Karatayeva, Lili Hegedus, Arindam Bhattacharjee, Eszter Nemeth, Adam Poti, Lorinc Pongor, Gabor Juhasz, David Szuts, Peter Burkovics

**Affiliations:** 1 Institute of Genetics, HUN-REN, Biological Research Centre, Szeged, Hungary; 2 Present Address: Developmental Biology Group, Agharkar Research Institute, Pune, India; 3 Institute of Molecular Life Sciences, HUN-REN Research Centre for Natural Sciences, Budapest, Hungary; 4 Cancer Genomics and Epigenetics Core Group, Hungarian Centre of Excellence for Molecular Medicine, Szeged, Hungary; 5 Doctoral School of Biology, Faculty of Science and Informatics, University of Szeged, Szeged, Hungary

**Keywords:** G-quadruplex, PhenDC3, Genome stability, Mitophagy, Genome, Genomic instability, Mitophagy, DNA

## Abstract

Guanine-rich DNA sequences have a propensity to form G-quadruplex structures. These structures play several important biological roles and are potential targets for anticancer drugs. However, no G-quadruplex-stabilizing agent has yet been approved for clinical use. Given that G-quadruplex stabilization is quite promising as a mechanism for novel anticancer therapies, it is crucial to elucidate its effects on healthy human cells. In our study, we modeled a potential human treatment using G4 -stabilizing agents and analyzed their effects on genome integrity, transcriptomic changes, and mitochondrial function focusing on non-cancerous cells to predict potential side effects of such treatments. We found that G-quadruplex stabilization does not compromise genome integrity. However, it can induce persistent alterations in the transcriptomic profile of human cells, including genes encoded on the mitochondrial genome. Notably, certain G-quadruplex-stabilizing agents triggered mitophagy in both human cells and *Drosophila melanogaster*. In summary, our findings indicate that while G-quadruplex stabilization does not cause genome instability, it may pose potential risks due to its long-term effects on transcription and its ability to induce mitophagy. Therefore, we recommend that all potential drug candidates be thoroughly evaluated for their ability to induce mitophagy and to promote cancer formation in animal models prior to clinical trials.

**Supplementary Information:**

The online version contains supplementary material available at 10.1038/s41598-025-04791-x.

## Introduction

Various secondary DNA structures can occur throughout the genome during DNA transaction processes (i.e., hairpins or cruciforms during replication, DNA triplexes) or can exist as its regular components (G-quadruplexes, Z-DNA, i-motif). At the same time these structures are documented to cause genomic instability through stalling the replication fork and deregulation of their formation or resolving is linked to diseases. The existence of such structures that impose a risk of rearrangements on the genome and can potentially jeopardise its integrity is quite a perplexing matter, which is worth exploring in the search for new therapeutic applications.

One of the most abundant non-canonical DNA structures is DNA quadruplex with more than 700,000 putative G4 sequences identified in the human genome^1–3^. Due to the ability of guanosine to self-associate through Hoogsteen hydrogen bonding, stretches of guanines can form a stable non-canonical structure consisting of guanine tetrads that can stack on top of each other and are further stabilized by a cation. G4 structures can be found across various species^3^ with their genomic locations being highly conserved^4^ and appear to have important biological functions in a living cell, such as gene expression regulation, chromatin organisation and epigenetic regulation, replication initiation and telomere formation^5,6^. Therefore, it is critical for a cell to be able to replicate them precisely and efficiently. An imbalance between formation and resolving of G4 structures could also have an implication in cancer as can be seen in the study by Biffi et al.^7^. This study demonstrated an elevated number of G4 structures in stomach and liver cancer cells, which could point at the dysregulation of DNA quadruplex processing in some types of cancer. G4 structures were found to be enriched at nucleosome-depleted highly active promoter regions^8^ of human genes with about 50% of them being promoters of proto-oncogenes^9^, as well as tumour suppressors and somatic genes linked to carcinogenesis (though in a smaller proportion to onco-genes promoters)^10^. For instance, the *c-MYC* promoter, which is upregulated in about 70% of tumours^11^, is enriched for G4 structures^12^.

Due to the diverse regulatory roles of G-quadruplex structures several G4 binding ligands were developed^13,14^. Recognition that impaired processing of G-quadruplex structures can contribute to cancer development has prompted the identification of several G4-binding ligands with therapeutic potential^15^. Even though the majority of these compounds bind a wide range of G4 structures, some of them show topological selectivity, for example Telomestatin preferentially binds to the antiparallel basket-type intramolecular G-quadruplex structures formed by human telomeric DNA^16^. CX-5461 stabilizes G-quadruplex structures formed by various sequences, including human telomeric DNA, c-MYC, and c-KIT1 promoters, and molecular dynamics simulations suggest that it binds to these G4 structures with varying affinities, showing the highest binding energy for human telomeric G4s^17^. RHPS4 shows a preference for hybrid and antiparallel G4 structures, particularly those formed by human telomeric sequences^18^. Although the overall structure of the G-quadruplex is similar, they also show a considerable sequence heterogeneity, therefore, binding affinity of different G4 ligands exhibits strong alteration on different G4 sequences^19^. Recently it has also been observed that G4 structures can multimerize to form higher order structures, playing a role in certain physiological processes and disease (reviewed in^20^), which adds another level of complexity to the topological targeting of G4 binding molecules. Hence, various G4 biding ligands could elicit to some extent different effects in vivo due to having unique G4 binding spectra in addition to the overall G4 binding properties. Moreover, it has been shown that different cell lines/cell types G4 formation patterns are distinct^21–23^. Therefore, it is evident that the G4 stabilization could have a broad range of effects depending on the type of G4 binding ligand and the cell type.

There is evidence that stabilization of DNA quadruplexes can cause genome instability under certain circumstances, for instance when a component of DNA repair machinery or a helicase implicated in G4 resolving is absent^24–26^. This phenomenon might either contribute to cancer development or induce enough DNA damage in cancer cells to trigger checkpoint activation and apoptosis, which is especially beneficial in case of cancers that are deficient in DNA repair. For example, this approach proved to be efficient in BRCA1 or BRCA2 deficient cancer cells and patient-derived xenografts (HR and NHEJ deficient cancers): a study by Xu et al.^27^ demonstrated that G4-stabilizing fluoroquinolones CX-5461 and related compound CX-3543 induce replication dependent damage and cell death in these cells. Other studies observed increased DNA damage, impaired telomere function and checkpoint activation upon stabilization of G4 structures^28–30^.

Another aspect of using G4-stabilizing agents as cancer therapy that should be taken into consideration is their possible effect on mitochondria. Mitochondrial DNA consists of a purine rich (G and A) heavy strand and a pyrimidine rich (C and T) light strand^31^. Since the high guanine content of the heavy strain of the mitochondrial genome, it can form G-quadruplex structures which have a role in various biological processes^32^. Mitochondrial DNA is packed into structures called nucleoids; unlike the nuclear genome there are no histones, however, other proteins physically associated with mtDNA were detected (reviewed in^33^). There is some evidence of DNA repair mechanisms in mitochondria, such as base excision repair, that competes with damaged DNA degradation and somewhat inefficient double strand break repair which are thought to be at least partially responsible for the emergence of deletions in mtDNA (reviewed in^34^). Mitochondrial genome is also rich in repetitive guanine stretches that are able to form G4s, with the density of these regions being several times higher compared to the nuclear genome^35^. G4 structures in the mitochondrial genome can serve various functions such as transcription termination at CSBII on the polycistronic transcript^36^. G4 structures in the mtDNA might also contribute to deletions and other mutations: an increase in mutation rate and, hence, genetic variation was observed in these regions^37^. Considering a less efficient DNA repair and the tendency of cells to remove dysfunctional or superfluous mitochondria, stabilization of DNA quadruplexes might affect the number of mitochondria in G4 ligand treated cells.

Since quite a significant proportion of the human promoters harbor at least one quadruplex structure, one would expect a change in the transcription not only in the oncogenes, but also on a global level. Indeed, a study by Halder et al.^8^ demonstrated that treating cells with PhenDC3 and 360 A, which bind G4 structures with high specificity, affected the transcriptome of HeLa S3 cell line. Considering the possibility of genomic instability triggered by stabilizing G4 structures, as well as their possible regulatory role in the expression of genes involved in cancer development^38^, we aimed to investigate the mutagenic potential of stabilizing these structures on the whole human genome and to explore the effects of this treatment on human cells. PhenDC3, a potent and highly selective G4 targeting ligand was selected to perform treatments of the primary cell line RPE-1 TP53^−/−^, with the p53 knockout being necessary in order to decrease cell senescence during treatment.

Since G4 stabilization is a potential and very promising new strategy for cancer therapy, it is important to elucidate the overall effect of G4 stabilization on human cells. Therefore, we analyzed how G4 stabilization affects nuclear genome stability, the transcriptional pattern of the treated cells as well as the stability and function of mitochondria. Overall, we found that G4 stabilization using PhenDC3 does not affect the stability of the genome, but it has a serious impact on the transcriptome and the mitochondrial function.

## Results

### Stabilization of G4 structures does not increase the spontaneous mutation rate

To examine how G4 stabilization affects the genome stability we used a p53-deficient version of the hTERT-immortalized retinal pigment epithelial cell line RPE-1. Since the absence of the p53 this cell line cannot go into apoptosis, therefore this cell line is useful to establish homologous recombination associated genome instabilities as described previously^39^. Since different G4 binding ligands has different binding spectra against the different G4 structures (19), we used PhenDC3 as a model G4-stabilizing agent, developed by De Cian et al.^40^, which showed wide G4 binding spectra, high specificity and high stabilization of G4 structures as well as blocking G4 unfolding by FANCJ^41^ and Pif1 helicases^42^.

**Fig. 1:**
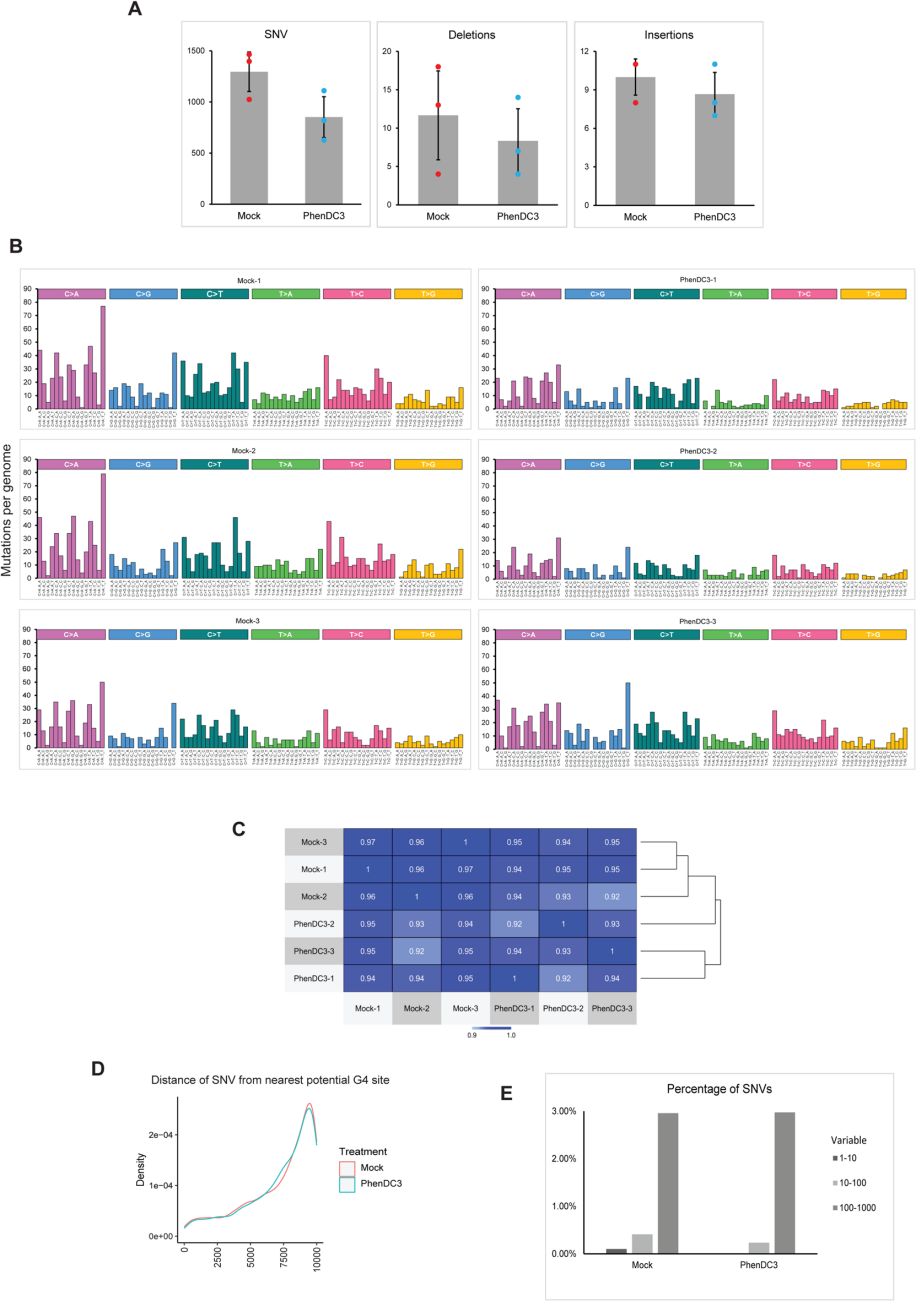
Cyclic PhenDC3 treatment does not increase the mutational rate. **A** Graphical representation of the amount of SNVs, small insertions, and deletions. **B** Mutational signatures in the control and PhenDC3 treated samples. **C** Representation of mutational profile similarities of treated and untreated samples by pairwise cosine similarity values. **D** Representation of the distance of SNV from the nearest potential G-quadruplex forming site. **E** Graphical representation of the percentage of SNVs from the nearest potential G4 sites.

The detailed mechanism of the G4 structure replication has not yet been fully elucidated. Although several publications suggested DNA polymerases which potentially contribute in G4 replication in vitro^43–45^, the in vivo contribution of a DNA polymerase has not been proved yet. Therefore, it is possible that the prolonged existence of G4 structures induces mutagenic bypass processes. We did not observe any substantial differences in the number of single nucleotide variations (SNVs) arising during the culture period from the whole genome sequencing results of mock and PhenDC3 treated RPE-1 cell lines (Fig. 1A). According to the mutational spectra, the most frequent mutations are C > A transversions (Fig. 1B), which are common for cells grown in cultures. C > A changes could be explained by the defects in the repair of 8-oxo-guanines^46^. Cells growing in cultures are often a subject to oxidative stress due to the fluctuations in oxygen levels during incubation and handling; moreover, stretches of guanines are particularly prone to oxidation^47^. This result is fitting well with the previously published study on the RPE-1 TP53^−/−^ cell line^39^, however, due to a longer period of growth, the control samples in the present experiment accumulated a slightly higher number of mutations. C > T mutations can be a result of deamination of unstable 5-methylcytosine to thymine at CpG islands which occurs spontaneously in vertebrate genomes^48^. The extent of the mutational profile similarities of treated and untreated samples are further confirmed by the pairwise cosine similarity values which are higher than 0.9 across all samples (Fig. 1C). No significant differences were observed for short deletions and insertions either (Fig. 1A). SNVs, as well as short indels usually arise in the genome due to replication errors, so the short indels that we observed in the treated and mock-treated samples can be attributed to natural mutational processes in a living cell. In order to observe minor effects on the mutability of G4 sites and their close surroundings, we determined the nearest G4 formation prone site to all SNVs using pqsfinder. We found that the distribution of the distance is similar for the mock and PhenDC3 treated samples, and the percentage of mutations in the closest proximity of potential G4 sites is slightly lower in case of PhenDC3 treatment indicating no mutagenic effect directly at the potential G4 sites (Fig. 1D, E).

Based on these results, we concluded that the stabilization and prolonged persistence of G4 structures did not result in increased base substitution rate under our experimental conditions.

### Stabilization of G4 structures does not affect large scale genome stability

**Fig. 2:**
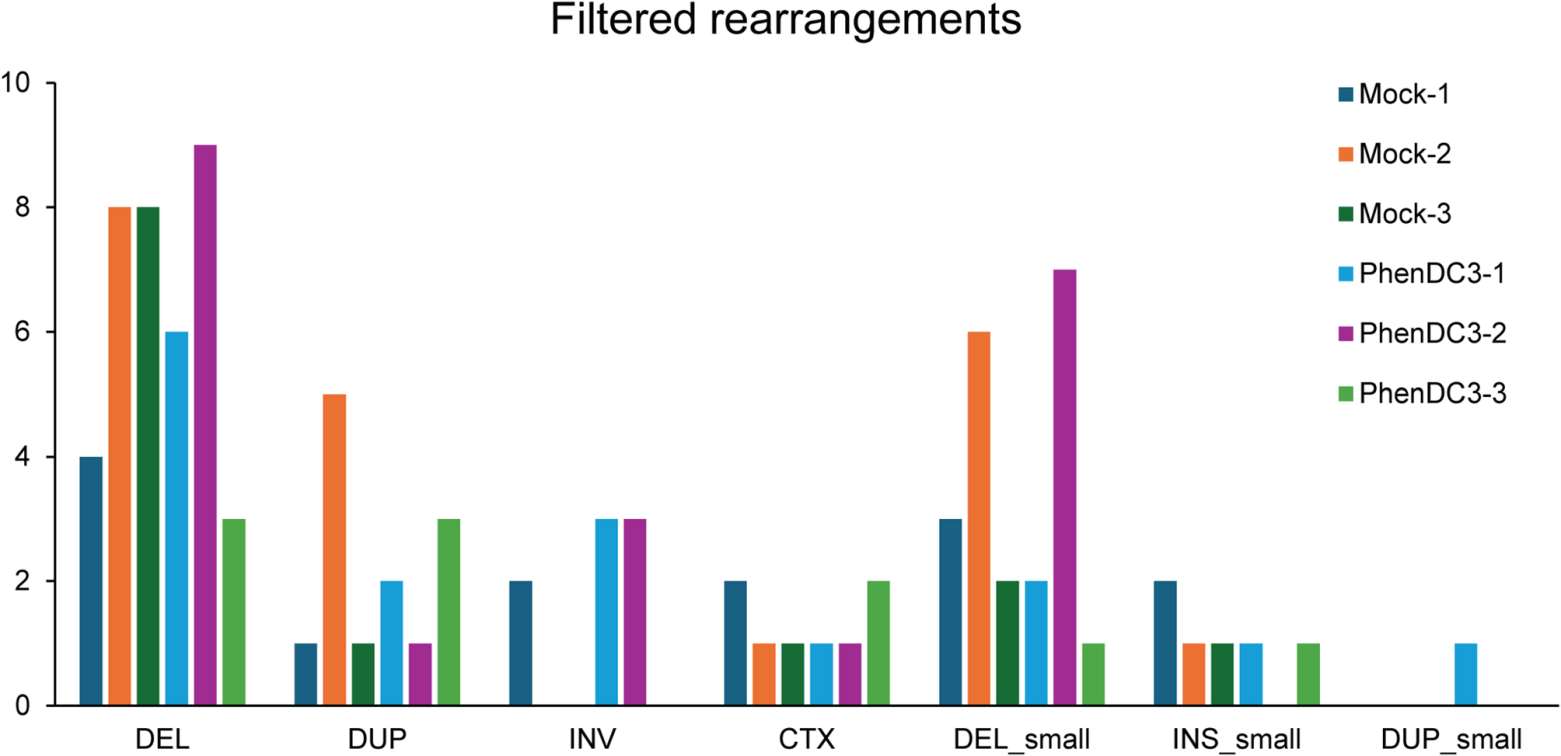
Cyclic PhenDC3 treatment does not induce genome instability. Graphical representation of large and small deletions, duplications, inversions, and gross chromosomal rearrangements were shown for each sequenced sample.

It is known that prolonged persistence of G4 structures could lead to replication fork stalling, which can in turn induce DNA double strand breaks (DSB)^29^. The repair of DSBs could result in genomic instability such as insertions, deletions or chromosome rearrangements. We performed a breakpoint analysis based on structural variation calling to detect large deletions and insertions, inversions and chromosome translocations, and found no significant difference in the numbers between the control and PhenDC3 treated samples. Assessment for large rearrangements across all the samples also show the same pattern with deletions of longer stretches of DNA being the most prevalent over other mutations (Fig. 2). The absence of increased mutational rate or genomic instability in the PhenDC3 treated samples can be due to the fact that the cell line used for the study did not have any defects in the DNA repair machinery and it appears that stabilization of the G4 structures can be successfully overcome by the cell’s mechanisms that resolve DNA quadruplexes under normal circumstances.

### Effect of G4 stabilization on global transcriptome

G-quadruplex formation potentially regulates transcription, because the G4 forming sequences are prevalent near the transcriptional start sites^38^. Since G4-stabilizing agents are being developed for therapeutic reasons it is important to know their possible effect on the transcription profile of healthy human cells, therefore, we investigated the effect of G4 stabilization on the transcriptional profile of treated p53 deficient RPE-1 cells.

**Fig. 3:**
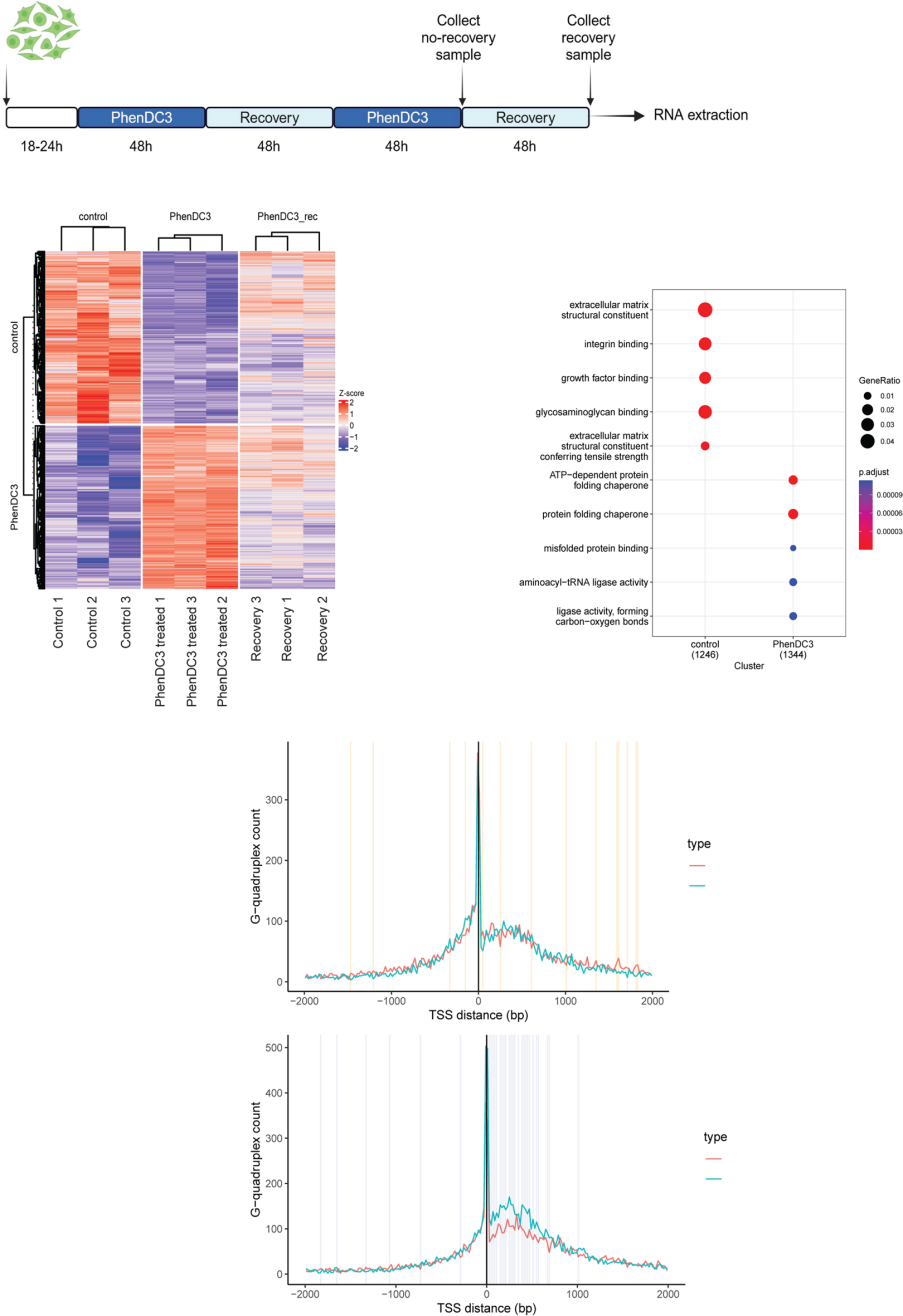
G4 stabilization affects the gene expression pattern of the human RPE-1 cells. **A** Graphical representation of the experimental set-up of the transcriptomic analyzes. **B** Heat map of the differentially expressed genes in control, PhenDC3 treated and recovery samples. **C** Graphical representation of functions of the differentially expressed gene. **D** Mapping of G4 prone regions in the differentially expressing genes environments. G4 prone regions are associated with the transcription start point. Their orientation is stochastic in the case of upregulated samples, but in the case of downregulated genes contain G4 prone sequence significantly frequently on noncoding DNA strand.

Although previous studies have reported that G4 stabilization exerts certain effects on the transcriptome profile of the treated cells, we employed a specialized experimental setup to model a patient treatment protocol. We used cyclic treatment with PhenDC3 and analyzed the transcriptomic profile of the cells, after the treatment and after a two-day recovery time (Fig. 3A). As we expected, the expression of several genes changed after the cyclic treatment with PhenDC3. In our study the expression of 4612 genes was altered: 2316 genes were upregulated, and 2296 genes were downregulated after PhenDC3 treatment (Fig. 3B). The functions of the affected genes are very diverse (Fig. 3C). We must note here that we used the same RPE-1 TP53^−/−^ cell line that we used in the genome stability assay, which can significantly affect the gene expression profile compared to wild type cells. We analyzed how the localization and orientation of G4 prone DNA regions was affecting the transcriptional changes after PhenDC3 treatment. The G4 prone structures (identified by pqsfinder) were found to be highly enriched around the transcription start site of the differentially expressed genes (Fig. 3D). Analyzing the downregulated genes after PhenDC3 treatment, we found G4 prone sequences on the coding and noncoding strand with equal frequency. But the G4 prone regions were significantly enriched on the noncoding strand downstream and in close proximity to the upregulated genes transcription start site (Fig. 3D).

**Fig. 4:**
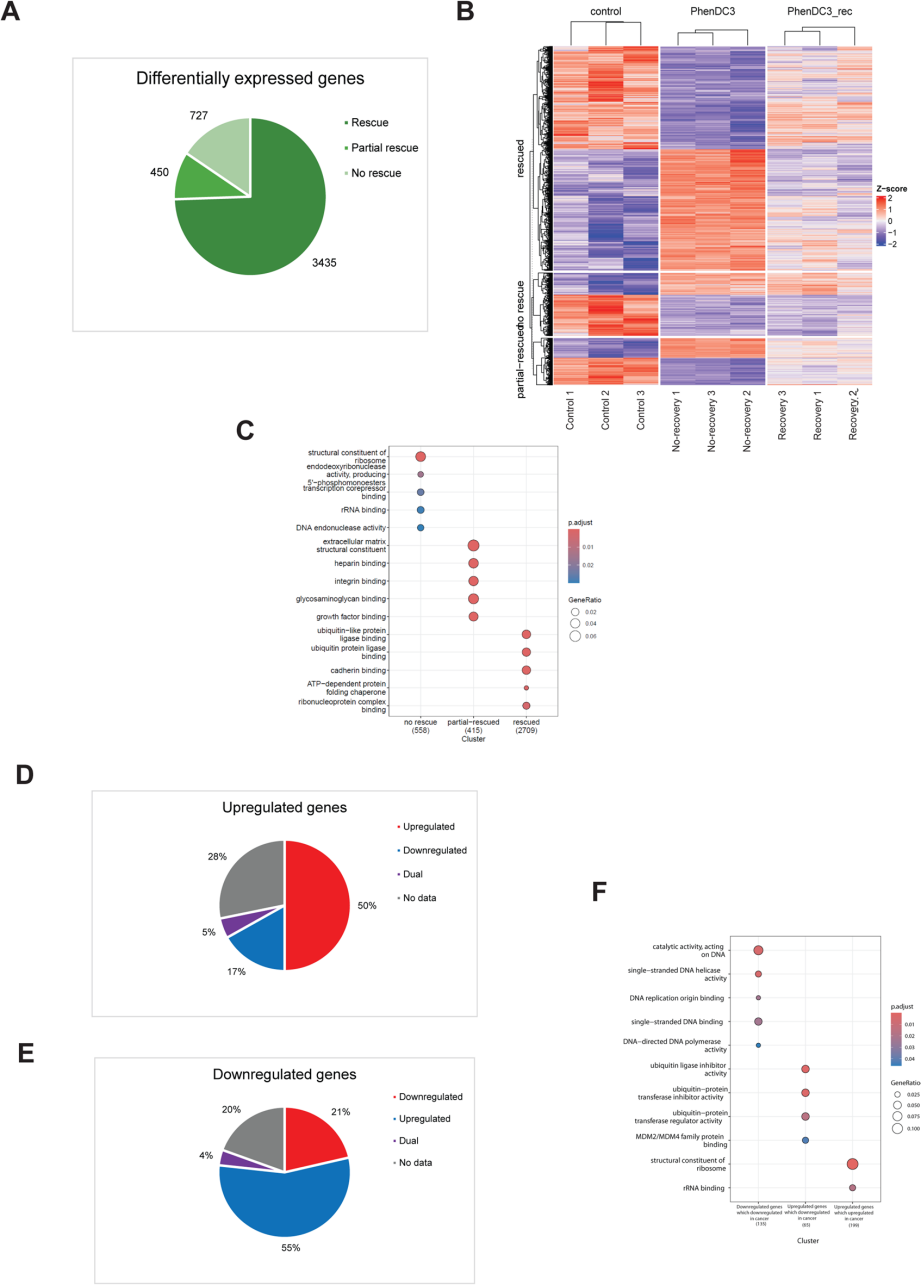
G4 stabilization has long term effects on the gene expression pattern of the human cells. **A** Graphical representation of the changes in the gene expression pattern after 48 h of the ending the PhenDC3 treatment. **B** Heat map representation of differentially expressed genes grouped as recovered, partially recovered, and not recovered. **C** Graphical representation of functions of the differentially expressed genes. **D** Graphical representation of the distribution of upregulated genes in the relation of published expression level changes in cancer patient samples. **E** Graphical representation of the distribution of downregulated genes in the relation of published expression level changes in cancer patient samples. **F** Graphical representation of functions of the subgroups of no-rescue genes categorized on **D** and **E**.

Since we tried to understand the potential risks of a G4 stabilization treatment, we analyzed the transcriptome of the treated RPE-1 cells after the whole cycle of the PhenDC3 treatment (Fig. 3A). After two-day recovery time the expression of 3435 genes was rescued (Fig. 4A), which means that there was no significant difference in the gene expression levels between the control and the recovered samples. Surprisingly there were 450 transcripts that were only partially rescued. The number of these transcripts were significantly different compared to the control and the PhenDC3 treated samples too. The expression of 727 genes was not recovered after the two-day recovery period. In these cases, the expression levels were significantly different compared to the control samples, and their expression level were not significantly different from the PhenDC3 treated samples (Fig. 4A and B, Supplementary Table 1). This finding suggests that the prolonged G4 stabilization not only transiently changed the gene expression profile of the human cells, but it can cause long term effects after the treatment is finished.

We further analyzed the no-rescue genes, and gene ontology analysis revealed their involvement in transcription, translation and DNA repair (Fig. 4C, Lane 1). Surprisingly in this subgroup the upregulated genes were overrepresented comparing to the PhendDC3 treated samples, where the numbers of up-and downregulated genes were almost equal. 465 of them were upregulated permanently. Interestingly, based on the previously published data 50% of them were described as upregulated genes in different types of cancers (Fig. 4D, Supplementary Table 2). Contrary to this observation, the 55% of 262 downregulated genes were also described as upregulated genes in different types of cancer and only 21% of the permanently downregulated genes were described as downregulated genes in various cancers. (Fig. 4E, Supplementary Table 2). Our gene ontological analysis on this subgroup demonstrated that the upregulated no-rescue genes, which are also upregulated in cancers, are primarily involved in ribosome biogenesis (Fig. 4F). The upregulated no-rescue genes, which are reported to be downregulated in cancer cells, are associated with the ubiquitination pathways. Meanwhile, the downregulated no-rescue genes, which are also downregulated in cancer, are mainly involved in DNA repair (Fig. 4F). Unfortunately, we were not able to categorize the downregulated no-rescue genes, which are upregulated in cancer. Since many fundamental cellular functions such as transcription, translation, DNA repair and ubiquitination, which is also an integral player in various regulatory mechanisms, were permanently changed, G4 stabilization therapy may pose potential risks to patients.

We also observed altered expression levels in the three well-established oncogenes. The expression levels of Bcl2 and KRAS were decreased after prolonged PhenDC3 exposure. The expression changes of Bcl2 were fully recovered after 48 h recovery following G4 stabilization, but the expression level of KRAS was only partially rescued. More interestingly, the expression level of MYC increased during and after the G4 stabilization, since it was categorized as a no-rescue gene (Supplementary Table 1). These data could further strengthen the risk of G4 stabilization as a chemotherapeutic strategy.

## G4 stabilization by PhenDC3 induces mitophagy

Mitochondria perform vital metabolic processes in the cell to provide it with energy, however, during respiration they produce reactive oxygen species that are potentially deleterious for the cell, as well as contain proapoptotic factors. The quality of mitochondria has to be well maintained since a defective/superfluous organelle can lead to cell death. For this reason, mitochondria possess several quality control mechanisms, and in case a mitochondrion is aged or terminally damaged it undergoes a selective autophagy process called mitophagy^49^.

**Fig. 5:**
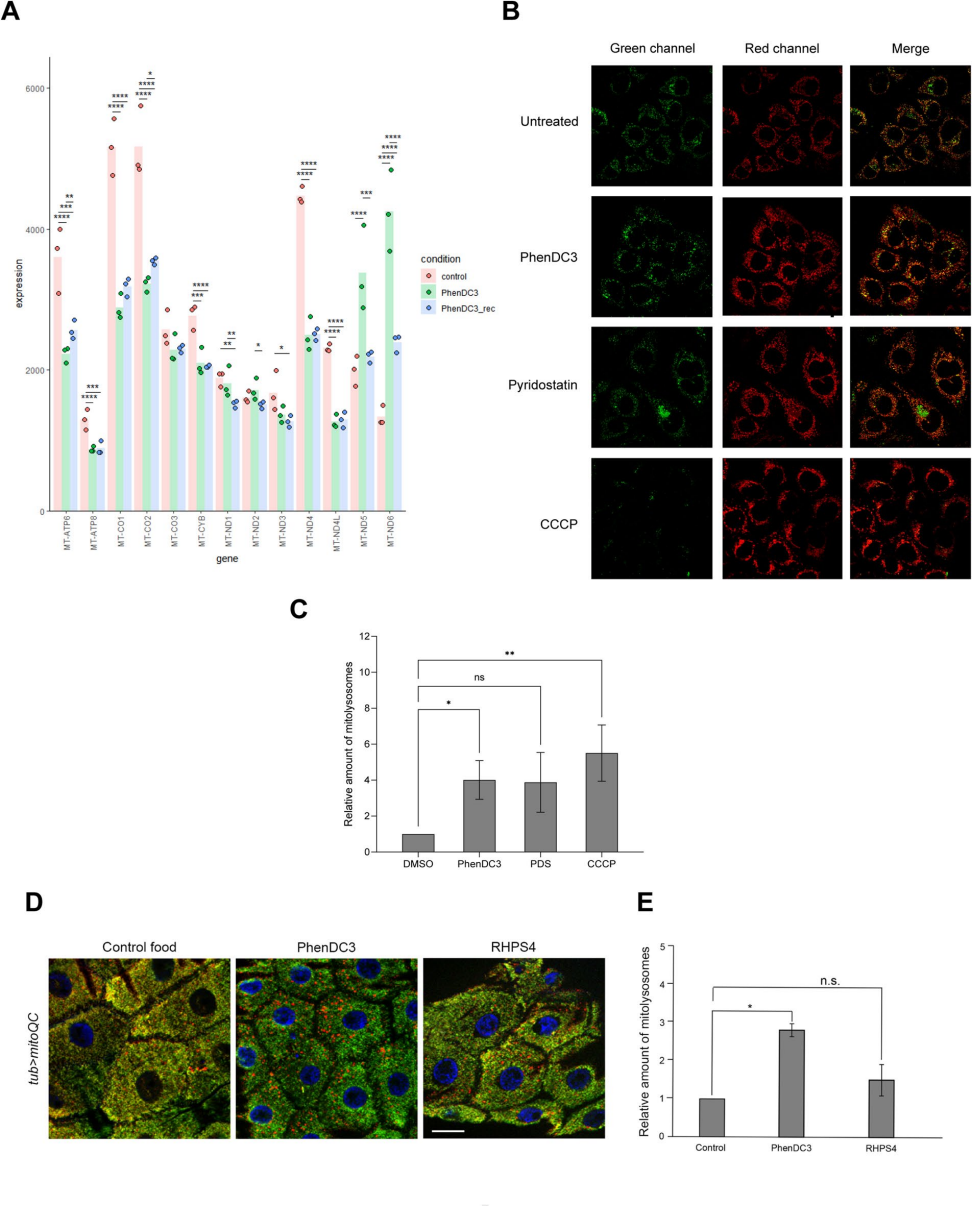
Certain G4-stabilizing agents induce mitophagy. **A** Gene expression profile of the 13 mitochondrial encoded genes in control, PhenDC3 treated, and Recovered samples. **B** PhenDC3 and Pyridostatin induce mitophagy in mtKEIMA expressing HeLa cells. **C** Graphical representation of the quantification of **B**. **D** Epidermal cells of *Drosophila melanogaster* larvae fed with PhenDC3 or RHPS4 as indicated. Red puncta (GFP^−^mCherry^+^) correspond to mitolysosomes. Scale bar = 20 μm. **E** Graphical representation of the quantification of **D**.

Our transcriptomic results demonstrated that the expression of all of the mitochondrially encoded protein coding genes was changed after PhenDC3 treatment (Fig. 5A). The expression levels of MT-ATP6, MT-ATP8, MT-CO1, MT-CO2, MT-CO3, MT-CYB, MT-ND1, MT-ND3, MT-ND4, and MT-ND4L were decreased, whereas the expression levels of MT-ND2, MT-ND5 and MT-ND6 were increased after G4 stabilization by PhenDC3. Additionally, the expression levels of these genes were not restored after the whole treatment was completed (Fig. 5A, Supplementary Table 1).

Aberrant mitochondrial transcription could lead to metabolic reprogramming, increased glycolysis, and decreased reliance on oxidative phosphorylation^50^. Additionally, it has been described that dysregulation of mitochondrial transcription factors (POLRMT, TFAM, TFB2M, and MTERF) could contribute to cancer progression and development^51–54^. Therefore, we generated mt-KEIMA expressing human HeLa cell line, to follow the mitophagy process in G4 stabilized human cells. To control our experiments, we leveraged the most widely used mitophagy inducer CCCP, a H + ionophore and uncoupler of oxidative phosphorylation in mitochondria that causes mitochondrial membrane depolarization and dysfunction of mitochondria, inducing PINK1/Parkin-mediated mitophagy^55^. Treatment of mt-KEIMA expressing HeLa cells with CCCP dramatically increased mitophagy as expected (Fig. 5B, C). Exposure to 10 µM PhenDC3 also resulted in a significant increase in mitophagy (Fig. 5B, C) as indicated by the elevated number of red fluorescence puncti, marking an increased number of mitochondria that have undergone autophagy. Treatment of mt-KEIMA cells with 10 µM Pyridostatin led to a mild, but not statistically significant, increase in mitophagy. Based on these findings, we conclude that G4 stabilization in human cells induces mitophagy.

Since a potential side effect of a drug that can induce mitochondrial dysfunction and mitophagy could be deleterious, we tested the mitophagy induction potential of the G4 stabilization on a living organism. We selected a widely used model for this experiment, the mitoQC expressing *Drosophila melanogaster*^56^. In this experiment we fed larvae with PhenDC3 or RHPS4 to induce mitophagy (Fig. 5D, E). As we expected, PhenDC3 was able to induce mitophagy similar to the positive control CCCP, but RHPS4 was not as efficient. This could be attributed to the fact that RHPS4 has a stronger preference to bind telomeric G4 over promoter G4^57^, whereas PhenDC3 has high affinity for both telomeric and promoter G4^58^.

In summary we can conclude that a subset but not all of the G4-stabilizing agents could induce mitophagy, therefore, a potential G4-stabilizing drug molecule has to be tested for this phenomenon.

## Discussion

There is a growing number of molecules, which stabilize G4 structures and have a potential application as chemotherapeutic agents. Therefore, it is crucial to understand, how G4 stabilization affects genome stability of a healthy cell. In the present study we aimed to assess the effects of G4 stabilization on the nuclear genome, global transcriptome and the state of mitochondria.

It was previously demonstrated that G4 structures can induce fork stalling during replication^43,59–64^, which in turn might lead to the appearance of mutations. It is possible that the replication of the G4-prone DNA regions is carried out by the replicative DNA polymerases such as the Polδ or Polε, or they are replicated via a translesion synthesis DNA polymerases similarly to the bypass of DNA damage. Considering possible contribution of the TLS polymerases, the replication of G4 structures with a prolonged persistence could be mutagenic. G4 mediated genomic instability is detectable in human cancer^65^. Ligand stabilization has been shown to locally induce genomic instability with a replicative origin in HeLa cells, at a specific locus^66^. The absence of an increased amount of point mutations, small deletions and small insertions in our study indicates that stabilized G4-forming DNA regions are replicated by an error-free high-fidelity mechanism in the wild type genetic background and do not increase genomic instability on a global genomic scale at a dose that causes transcriptomic changes.

Several studies have described that the G4 stabilization has a profound effect on the genome stability if any component of the G4 processing machinery is missing, but usually there was only a slight effect of the G4 stabilization in the wild type background^42,67–69^. In our study we were not able to detect a significant increase in the number of genome rearrangement events. Moreover, several studies have described increased point mutation frequency around G4 structures in different clinical samples from cancer patients^65,70^. In our interpretation, the function of G4-processing proteins is impaired in these cancer samples, but not in RPE-1 cells used in our study, leading to increased genomic instability. This finding demonstrates that without any failure in the G4 processing/replicating machinery the G4 stabilization alone cannot induce genome instability. This suggests that G4-stabilizing agents may pose a low risk of genome instability in clinical applications.

G-quadruplex forming sequences are prevalent in many promoter regions, therefore, changes in the stability of G-quadruplex structures have a profound effect on the gene expression profile of treated cells as described previously^71^. G4 stabilization could also lead to epigenetic changes in the cell genome due to the modifications in the methylation status of CpG island-containing promoter regions^72,73^, and this can in turn modify the activity of the histone modification enzymes^5^. Therefore, it is important to elucidate how a prolonged treatment using a G4-stabilizing agent (in this case PhenDC3) affects the transcriptome, and most importantly to analyze how the transcriptome behaves after the treatment is completed. For this reason, we treated human RPE-1 p53-deficient cells with PhenDC3 in a cyclic manner and analyzed the transcriptome profile of the cells at the end of the treatment and after two days of recovery time.

As we expected, the transcriptomic profile of the PhenDC3 treated (G4 stabilized) cells was dramatically changed compared to the control (DMSO treated) cells. Analyzing the distribution of potentially G4-forming motifs in the genomic sequences of the up- or downregulated genes, we demonstrated that the highest abundance of the G4-prone genomic regions is around the transcription start position^24,74^. We analyzed independently the sequence context of the up- or downregulated genes. In case of the downregulated genes, we found G4-prone sequences on the + and − strands with equal frequency after PhenDC3 treatment. In the genomic context of the upregulated genes the G4-prone regions were significantly enriched on the + strand downstream and in close proximity of the transcription start points.

However, our major interest was to examine the status of the transcriptome at the end of the treatment. Notably the 48-hour recovery time was not sufficient for the full restoration of the transcriptome. We were able to identify three different groups of genes in the recovery sample. Around 74% of the transcripts were restored to the control level (not significantly different from the control sample), but 26% still showed an altered gene expression profile. One subgroup of them was categorized as partially recovered, meaning that their expression level is significantly different from both the control and the PhenDC3 samples; this subgroup contains 38% of these transcripts. The second subgroup comprises of the no-rescue genes (62%), which expression levels are significantly different from the control sample, but not significantly different from the PhenDC3 samples. This persistent alteration may be due to RNA stability or epigenetic modifications affecting gene expression. Although the exact mechanism remains to be elucidated in future studies, it is likely that these changes contribute to the long-term transcriptome shifts and, therefore, impact the overall state of the cell. This could be a potentially dangerous off-target effect of G4 stabilization in clinical applications.

It is worth pointing out that around 50% of the upregulated genes, which were categorized as “no-rescue”, were also found to be upregulated in various types of cancers. This does not necessarily mean that cells after PhenDC3 treatment are in a „cancer-prone” transcriptional state, because in the case of downregulated genes, the majority of the transcripts (50%) were also described as upregulated transcripts in different cancer types. However, increased expression of certain cancer marker genes could be attributed to the changes in the overall stability of G4 structures during cancerous transformation. Moreover, we demonstrated that the cancer related no-rescue genes are implicated in ribosome formation, transcription, DNA repair and protein ubiquitination. Therefore, it is highly likely that the essential functions of the cell could be disrupted after G4 stabilization treatment is completed. This highlights both the critical role of G4 dynamics and the potential risks associated with using G4 stabilization as a chemotherapeutic strategy.

We also observed that the expression levels of the 13 mitochondrially encoded protein coding genes were dramatically changed by G4 stabilization. This could be explained by the fact that 12 out of the 13 genes are coded on the heavy (H) strand of the mitochondrial genome, which has a high GC content resulting in plenty of potential G4-prone sequences. Only MT-ND6 is coded by the light (L) strand^75,76^. Nonetheless, the MT-ND6 expression is also affected by PhenDC3 treatment. Majority of the mitochondrial encoded genes were downregulated after PhenDC3 treatment except for MT-ND5 and MT-ND6. The global downregulation of the 11 mitochondrially encoded genes could be caused by the less efficient transcription of the H chain. Additionally, processing of the polycistronic transcripts as well as the surveillance system regulating levels of the mitochondrial transcripts could also be affected by G4 stabilization resulting in an increased amount of these transcripts. The increased amount of MT-ND6 transcript, which is known to contain four G4 forming sequences^77^, could be the result of compromised melting of G4 structures by GRSF1^78^ and hence inefficient degradation of the mRNA. The same mechanism could also lead to an increased amount of ND5, which antisense long-noncoding RNA is also bound by GRSF1 to positively regulate its decay^78^. These transcripts, that are both encoded on the L strand, were found to be upregulated when the mitochondrial degradosome is inactivated^78^. Another possibility is that the elevated transcription initiation activity of the mitochondrial genome could compensate for the inefficient function of the oxidative phosphorylation, and hence decreased ATP concentration^79^.

Interestingly all the 13 mitochondrial transcripts were categorized as „no-rescue”, which suggests that the mitochondrial function is also significantly impacted during and after G4 stabilization treatment. Since mitochondria with functional abnormalities are often removed by mitophagy, a special form of autophagy, we tested whether G4 stabilization could induce mitophagy^80^, and indeed we observed increased numbers of mitolysosomes in PhenDC3 and Pyridostatin treated cells, however in the case of Pyridostatin the increase was not significant. As we expected, treating mtKEIMA expressing HeLa cell line with these agents increased the number of mitolysosomes. This dramatic effect of PhenDC3 treatment propounds a potentially dangerous side effect of G4 stabilization in clinical applications by depleting the healthy cellular pool of mitochondria.

Given the potential of G4-stabilizing agents to induce mitophagy and pose significant risks during treatment, we evaluated this effect on a living model organism. We used *Drosophila melanogaster*, because basal and chemically induced mitophagy across both larval and adult tissues have been well-characterized^56^. Similar to our cell-based experiment, feeding PhenDC3 to *Drosophila* larvae strongly induced mitophagy in the epithelial cells, producing an effect comparable to that of CCCP (not shown). This result indicates that oral administration of G4-stabilizing agent can induce mitophagy in normal somatic cells, highlighting a potential risk associated with of this type of anticancer treatment. Therefore, the most important question at this point is the following: is there any G4-stabilizing agent which does not induce mitophagy? Recently, a novel reporter system has been built, in which the mitochondrial G4 structure was stained by targeting BG4 antibody expression to mitochondria. In this study RHPS4 was used as G4-stabilizing agent, and it did not induce mitophagy^81^. RHPS4 inhibits telomerase activity but also exhibits mitochondrial localization and inhibits mitochondrial transcription elongation^82,83^. To verify this published result, we fed RHPS4 to *Drosophila* larvae. In our experiment RHPS4 did not induce mitophagy in the epithelial cells of *Drosophila* larvae, presenting evidence that not all of the G4-stabilizing molecules induce mitophagy. Since both PhenDC3 and RHPS4 affect the expression level of mitochondrially encoded genes, but only PhenDC3 was able to induce mitophagy, it suggests that mitophagy by some G4-stabilizing agents is not caused by changes in the levels of mitochondrial mRNA and/or protein.

In summary, our findings demonstrate that prolonged G4 stabilization does not cause genomic instability in the nuclear genome in RPE-1 TP53^−/−^ cell line. However, it leads to long-term changes in the transcriptomic profile of the treated cells. Additionally, certain G4-stabilizing agents can induce mitophagy which poses a significant safety concern for their use as a chemotherapeutic agent. Therefore, we recommend that all G4-stabilizing compounds be evaluated for mitophagy-inducing potential during the early stages of drug development to enable the early identification and exclusion of potentially harmful drug candidates.

## Materials and methods

### Cell lines and culture

The human hTERT RPE-1 TP53^−/−^ cell line was maintained in DMEM F-12 medium supplemented with 10% heat-inactivated fetal bovine serum and 1% penicillin/streptomycin and cultured at 37 °C with 5% CO_2_.

### Generation of ancestral and descendant clones

RPE-1 TP53^−/−^ ancestral and descendant clones were obtained by diluting cells to a concentration of about 30 cells per 10 ml of media, and the cells were seeded into 96-well plate from the mixture. Cells of the selected clone were then treated in the following manner: mock treatment with 0.5% DMSO, cyclic treatment − 10 µM PhenDC3 alternating with 0.5% DMSO every 2 days. The total time of treatment was 60 days (15 cycle). The last passages of treated cells were again diluted to obtain individual descendant clones, which were expanded to a density of about 1.8-2 × 10^6^ cells to extract genomic DNA.

### WGS and data analysis

Genomic DNA was extracted by lysing cells in 200mM NaCl, 20mM EDTA, 40mM Tris pH 8.0, 0.5% SDS, 0.5% 2-mercaptoethanol and incubating at 55 °C overnight and precipitating DNA from the mixture by ethanol next day. Library preparation and whole genome sequencing was performed by Novogene, Beijing China, using Illumina HiSeq 2500 (2 × 150 bp paired end format). One ancestral clone and three samples from the treated and untreated samples were sequenced.

Adapters were trimmed from raw sequencing reads using Cutadapt^84^. Reads were aligned to the human genome reference GRCh38/hg38 with the Burrows-Wheeler algorithm^85^ and post-processed with the IndelRealigner tool of the Genome analysis Toolkit (GATK, version 3.8)^86^. SNVs, small deletions and insertions were called using Isomut^87^; this method allows for simultaneous comparison of up to 30 samples with a detection rate of ~ 90% at 20–30 × coverage. Standard parameters were used: base quality filter 30, minimum mutated allele frequency 0.2, minimum coverage of the mutated sample 5, minimum reference allele frequency of all the other samples 0.93. Hits were post-filtered with the minimal criteria for SNV score 2.6, and for indels 3.8 To analyze the large genomic rearrangements, we used GRIDSS v2.8.3^88^. Events were post-filtered based on a panel of normals.

Potential G4-forming sequences were identified using pqsfinder^89^. DNA sequence was analyzed in a ^+/−^ 10 kbp proximity of all SNV sites. All sequences with a pqs score > 20 were considered as possible G4 forming site.

### RNA sequencing

Cells for RNA sequencing were treated with DMSO or 10 μm PhenDC3 for 2 cycles (1 cycle of treatment lasted 2 days followed by 2-day recovery) with or without final recovery (2 days without treatment) in 10 cm dishes. After designated treatment methods, cells were collected, snap-frozen and kept in − 80 °C before RNA extraction. RNA extraction, library preparation and RNA sequencing were performed by DeltaGene, Szeged, Hungary, using Illumina HiSeq 2500 (2 × 150 bp paired end format). Three samples from each group were sequenced.

### Transcriptome analysis

Sequencing read quality QC was performed with *FastQC* (v0.12.1). Reads were mapped to the hg38 human genome version using the gencode v45 annotation using the *STAR* aligner^90^ (v2.7.11a) in two-pass mode based on the parameters described in the “best recall at base and read level” as shown in supplementary Table 37 of^91^. Gene expression raw read counts were obtained with *featureCounts*^92^ from the subread package (v2.0.2). Normalized TMM-FPKM expression values were calculated using the *edgeR*^93^ package (v4.4.0) in R. Differential expression analysis was calculated using *DESeq2*^94^ (v1.46.0) using the raw read counts. Expression heatmaps were generated using the *ComplexHeatmap*^95^ package (v2.22.0) in R. Gene ontology analysis was calculated using the *clusterProfiler*^96^ package (v4.14.4) in R. The R version 4.4.2 was used for normalization, differential analysis and heatmap generation.

### Enrichment of G-quadruplex at gene promoters

G-quadruplex coordinates in BED format were obtained from the EndoQuad database^97^. For each analyzed gene, a 2 kb upstream and 2 kb downstream region surrounding the transcription start site was selected. These regions were intersected with the G-quadruplex BED file to identify overlapping features. The number of sense and antisense G-quadruplexes was then quantified for control and PhenDC3-associated genes by binning the window into 50 bp segments. Statistical differences in enrichment between PhenDC3 and control samples were assessed using a Poisson test in R.

### Generation of MtKeima expressing stable cell lines, and the measurement of mitophagy

MtKeima containing plasmid was a gift from Pan^98^. MtKeima cell line was created using 2nd generation lentiviral vectors in HeLa mCAT1 cells.

To measure the level of mitophagy, cells were plated onto 24 well plates on glass slides. After 24 h, cells were treated with 10 μm carbonyl cyanide 3-chlorophenylhydrazone (CCCP), 10 μm PhenDC3, pyridostatin, RHPS4, or DMSO (as a mock treatment) as indicated on the Fig. 4b for 24 h to determine the level of mitophagy after treatment in the cells. Cells were analyzed with Olympus Confocal LSM in both green and red channels to determine the level of mitophagy (increased level of red fluorescence) after inducing mitophagy in the cells. Microscopy images were analysed on ImageJ software to count the amount of mitolysosomes (i.e., mitochondria-containing lysosomes, counted as red punctate foci larger than 0.5 μm) in each sample.

### Drosophila mitophagy assay

The UAS-mito-QC Drosophila stock was obtained from Bloomington Drosophila Stock Center (#91641)^56^ and its expression was driven by tub-GAL4. L2 stage *tub > mitoQC* larvae were washed and transferred to standard yeast, cornmeal, agar food (control) or food containing PhenDC3 (50 µM) or RHPS4 (30 µM) for 24 h. Subsequently, the larval epidermis was dissected, fixed with 4% paraformaldehyde and stained with DAPI. Images were acquired on a Zeiss Axioimager M2 equipped with an Apotome module. After global brightness and contrast adjustments to remove background, the green fluorescence was subtracted from red and the remaining punctate signal (GFP^−^mCherry^+^) larger than 0.5 μm was counted as individual mitolysosomes per cell. Three independent experiments were performed with 5–6 larvae each.

## Electronic supplementary material

Below is the link to the electronic supplementary material.

Supplementary Material 1

Supplementary Material 2

Supplementary Material 3

## Data Availability

Data obtained for this study is available in binary alignment map format from the European Nucleotide Archive under study accession number PRJEB85998.
