# Supplementary material for: The effect of prolonged G-quadruplex stabilization on the functions of human cells
Source: Sci Rep. 2025 Jun 4;15:19699. doi: 10.1038/s41598-025-04791-x (PMC12137815; doi:10.1038/s41598-025-04791-x)
Supplement: Supplementary file 3 — Supplementary Material 3 [file 41598_2025_4791_MOESM3_ESM.docx]

**Supplementary table 1:** Categorized list of Differentially Expressed Genes. Differentially expressed genes were categorized as rescued, partial-rescue, and no-rescue genes based on the expression level changes in Mock, PhenDC3 treated and recovery samples.

**Supplementary table 2:** Relation of no-rescue genes to the published gene expression changes in cancer cells. Red color indicated similar changes in the experiment and in the published literature. Blue color indicated the opposite changes in our experiment and the published data from cancer cells. Purple color indicated contradictory data in literature. Black color indicated the absence of published data from cancer cells in the literature. PMID column contains the PMID number(s) of the appropriate publication(s) used for our categorization.
